# Prognostic risk factors in upper gastrointestinal perforation: the role of body composition parameters

**DOI:** 10.3389/fnut.2025.1651741

**Published:** 2025-09-05

**Authors:** Teng Zhu, Huayan Gu, Yuefeng Chen, Zeyuan Huang, Wandong Hong, Xiangjian Chen

**Affiliations:** ^1^Department of Gastrointestinal Surgery, The First Affiliated Hospital of Wenzhou Medical University, Wenzhou, China; ^2^Department of Trauma Surgery, The First Affiliated Hospital of Wenzhou Medical University, Wenzhou, China; ^3^Department of Breast Surgery, The First Affiliated Hospital of Wenzhou Medical University, Wenzhou, China; ^4^Department of Gastroenterology and Hepatology, The First Affiliated Hospital of Wenzhou Medical University, Wenzhou, China

**Keywords:** upper gastrointestinal perforation, prognosis, body composition, predictive model, risk factors

## Abstract

**Background:**

Despite medical advances, the prognosis for upper gastrointestinal perforation remained poor. The aim of our study was to identify predictors of adverse outcomes.

**Methods:**

We retrospectively analyzed laboratory data from patients with upper gastrointestinal perforation at the First Affiliated Hospital of Wenzhou Medical University (January 2021–December 2023), categorizing them according to septic shock, intensive care unit (ICU) admission, and postoperative complications.

**Results:**

Univariate and multivariate analyses of 200 patients with upper gastrointestinal perforation identified predictors of a poor prognosis: low muscle reserve (OR = 3.82, 95% CI 1.36–10.71, *p* = 0.011), high visceral fat area (VFA) (OR = 3.54, 95% CI 1.16–10.80, *p* = 0.026), and platelet-to-lymphocyte ratio (PLR) (OR = 1.01, 95% CI 1.01–1.01, *p* = 0.048) predicted septic shock. Sex (OR = 0.22, 95% CI 0.06–0.78, *p* = 0.020), high VFA (OR = 4.84, 95% CI 1.38–17.02, *p* = 0.014), prothrombin time (PT) (OR = 1.54, 95% CI 1.09–2.18, *p* = 0.014), and D-dimer (OR = 1.16, 95% CI 1.01–1.34, *p* = 0.048) influenced ICU admission. Meanwhile, surgical approach (OR = 7.82, 95% CI 1.94–31.57, *p* = 0.004), maximum perforation diameter (OR = 1.08, 95% CI 1.02–1.15, *p* = 0.013), and white blood cell (WBC) count (OR = 0.88, 95% CI 0.78–0.99, *p* = 0.039) were linked to postoperative complications.

**Conclusion:**

Our research found that the following factors were prognostic for upper gastrointestinal perforation: low muscle reserve, high VFA, PLR, sex, PT, D-dimer levels, surgical approach, WBC count, and perforation size.

## 1 Introduction

Upper gastrointestinal perforation is a common acute abdominal condition in surgical patients, characterized by an acute onset and rapid progression. Predisposing factors include disease factors, such as long-term chronic peptic ulcers, gastric tumors, and trauma. Drug factors include the long-term use of non-steroidal anti-inflammatory drugs or corticosteroids. Poor lifestyle habits, such as excessive eating, can also be a factor. In some cases, upper gastrointestinal perforation can cause septic shock, endanger the patient’s life, and result in a poor prognosis. Although surgery is currently the most reliable treatment for patients with upper gastrointestinal perforation, the prognosis for some patients remains unsatisfactory (1–3). Further research is imperative to explore the factors influencing prognosis, which may thereby contribute to optimizing perioperative management.

Numerous studies have confirmed the association between nutritional status and the prognosis of gastrointestinal perforation. Commonly used assessment indicators include serum albumin levels (4) and the prognostic nutritional index (PNI) (5). Current evidence indicates that preoperative serum albumin levels not only reflect a patient’s immediate nutritional status but are also associated with disease severity (6). Furthermore, it demonstrated the positive association between low albumin levels and mortality in peptic ulcer perforation through Miller MH’s meta-analysis (4). However, these nutritional assessment indicators are often difficult to obtain prior to emergency surgery. Consequently, body composition parameters, which can be obtained easily via CT scans, can serve as surrogate indicators of nutritional status. Numerous studies have reported strong correlations between body composition parameters and prognosis for various cancers (7, 8). Recent evidence has revealed that body composition was a risk factor for postoperative complications and a significant prognostic factor for abdominal surgery (9–15). Specifically, decreased skeletal muscle mass has been recognized as a critical factor (16–18), with reduced psoas muscle mass being independently associated with a poor prognosis in cases of lower gastrointestinal perforation (19). Nevertheless, research examining the impact of body composition on the postoperative prognosis of patients with upper gastrointestinal perforation remains scarce.

Therefore, this study aimed to investigate the prognostic factors in patients with upper gastrointestinal perforation by incorporating body composition analysis with the goal of developing a reliable clinical prognostic prediction model to provide individualized clinical guidance.

## 2 Materials and methods

### 2.1 Patients

This retrospective cohort study included consecutive patients who underwent surgery for upper gastrointestinal perforation at the First Affiliated Hospital of Wenzhou Medical University from January 2021 to December 2023. The inclusion criteria were as follows: (1) age ≥18 years, (2) definitively diagnosed with upper gastrointestinal perforation, and (3) underwent surgery for upper gastrointestinal perforation. The exclusion criteria were: (1) failure to undergo surgical treatment, (2) malignant tumor perforation, (3) underlying diseases that severely affected prognosis, and (4) did not have abdominal computed tomography (CT) scans before surgery in our hospital, or did not have other necessary clinical data. The study protocol was approved by the Ethical Review Board of the First Affiliated Hospital of Wenzhou Medical University.

### 2.2 Measurement of body composition parameters

Cross-sectional CT images of the third lumbar vertebra (L3) in the inferior direction were selected for body composition analysis. Skeletal muscles were separated from other tissues using a Hounsfield unit threshold range of −29 to +150, and tissue boundaries were manually outlined as needed. Muscles in the L3 region include the psoas, the erector spinae, the quadratus lumborum, the transversus abdominis, the external and internal obliques, and the rectus abdominis. To reduce measurement bias, a researcher who was blinded to patients and surgical characteristics was trained to identify and measure body composition using the sliceOmatic image processing system. The skeletal muscle index (SMI) was derived by normalizing the skeletal muscle cross-sectional area by height (m^2^). Skeletal muscle density (SMD) was quantified as the mean HU value within muscle areas. Adipose tissue compartments were defined as follows: subcutaneous fat area (SFA), using HU values of −190 to −30, and visceral fat area (VFA), using HU values of −150 to −50 (20). The visceral-to-subcutaneous fat ratio (VSR) was subsequently calculated. Low SFA was defined as <62.0 cm^2^ for females and <38.1 cm^2^ for males. High VSR was defined as ≥1.0579 for both sexes (21). Low SMI was defined as SMI <34.9 cm^2^/m^2^ in females and <40.8 cm^2^/m^2^ in males (22); low SMD was defined as SMD <28.6 HU in females and <38.5 HU in males (23). Both conditions were defined as low muscle reserve. High VFA was defined as ≥100 cm^2^ for both sexes, according to the diagnostic criteria for “obesity disease” established by the Japan Society for the Study of Obesity (JASSO) (24).

### 2.3 Data collection

The following data were collected: (1) Patient factors included age, sex, preoperative comorbidities, and BMI. (2) Preoperative factors included white blood cell (WBC) count, red blood cell (RBC) count, platelet count (PLT), lymphocyte count, C-reactive protein (CRP) concentration, prothrombin time (PT), international normalized ratio (INR), fibrinogen (FIB), activated partial thromboplastin time (APTT), D-dimer (D-D), platelet-to-lymphocyte ratio (PLR), neutrophil-to-lymphocyte ratio (NLR), and time from onset to anti-infective therapy and surgery. (3) Intraoperative factors included the cause of perforation, the perforation site, the maximum diameter of the perforation, and the surgical approach. (4) Postoperative factors included the Acute Physiology and Chronic Health Evaluation (APACHE) II score, the Sequential Organ Failure Assessment (SOFA) score, and the albumin level. (5) Outcome data included length of stay (LOS), intensive care unit (ICU) length of stay (ICU LOS), postoperative complications, septic shock, and postoperative length of stay. Septic shock is the most dangerous stage of sepsis progression. It is characterized by an uncontrolled systemic inflammatory response triggered by infection, leading to severe circulatory failure that is refractory to fluid resuscitation, along with clear evidence of tissue hypoperfusion. Specifically, it is defined as patients with sepsis who, after receiving adequate fluid resuscitation, still require vasoactive agents to maintain a mean arterial pressure (MAP) ≥65 mmHg, and have a serum lactate level >2 mmol/L. (25) All postoperative complications were classified using the Clavien–Dindo classification system (CD) (26), with CD grade II or above being defined as postoperative complications for the purposes of this study.

### 2.4 Statistical analysis

Qualitative outcomes were presented as percentages, while quantitative results were presented as median ± SD. Continuous variables with normal distribution were analyzed using the Student’s *t*-test, while those with non-normal (skewed) distribution were analyzed using the Mann–Whitney *U*-test. For categorical variables, the chi-square or Fisher’s exact test was used.

Logistic regression was used to assess the factors (*p* ≤ 0.05) identified in the univariate analysis within a multivariate model. Meanwhile, the odds ratio (OR) and 95% confidence intervals (CIs) were calculated. The cohort was randomly split into training and validation sets at a ratio of 7:3. Using backward regression, the training set was used to develop the logistic regression prediction model and construct the nomogram, while the validation set was reserved for internal validation. Using the receiver operator characteristic (ROC) curve, the C-index of the nomogram was calculated.

With SPSS version 26.0 software (SPSS Inc., Chicago, IL, United States), all statistical tests were performed. For statistical significance, a two-tailed *p*-value of less than 0.05 was set.

## 3 Results

### 3.1 Patients

**Figure 1:**
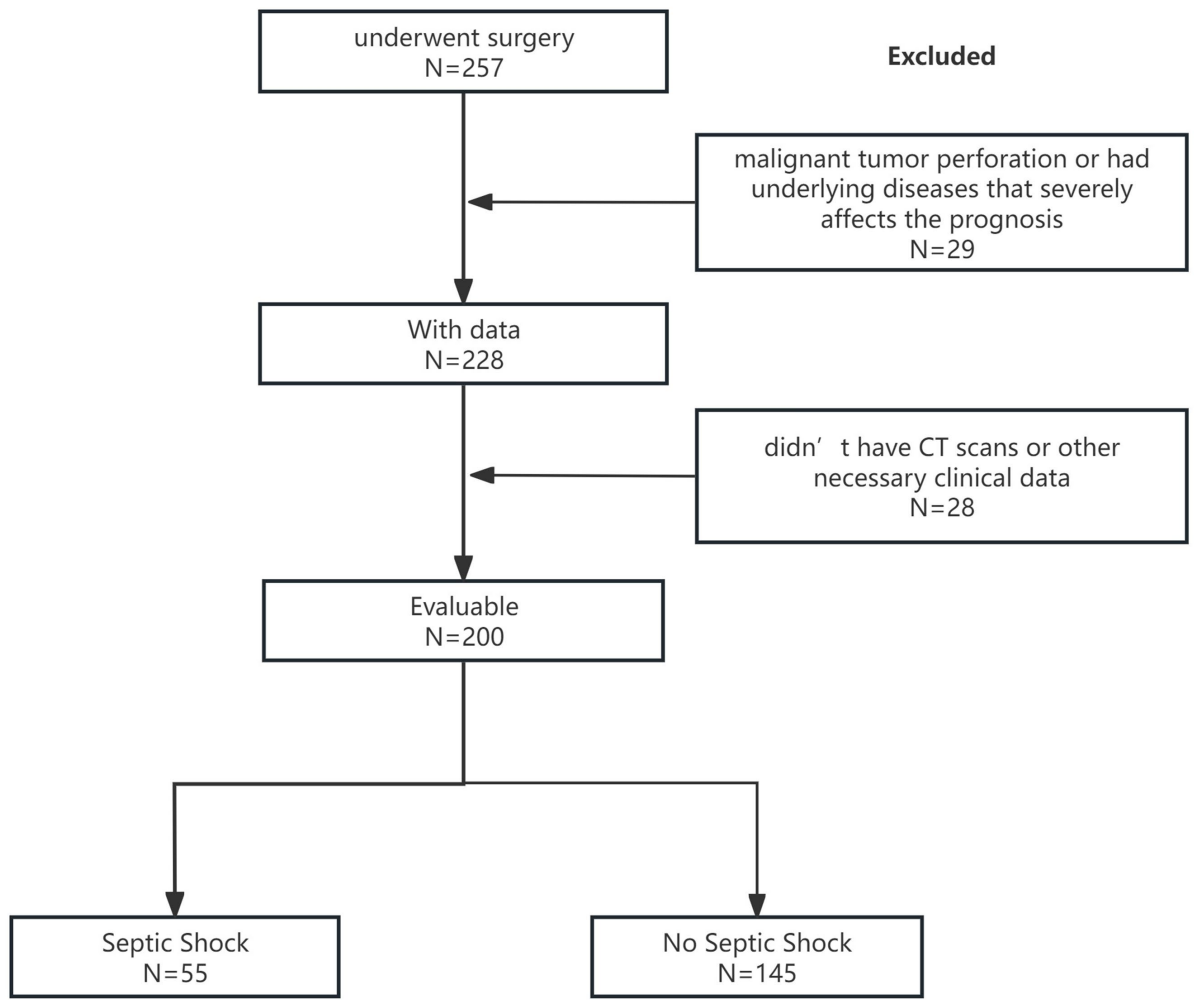
Study flow diagram.

Of the 257 consecutive patients who underwent surgery for upper gastrointestinal perforation at the First Affiliated Hospital of Wenzhou Medical University from January 2021 to December 2023, 29 patients were excluded for malignant tumor perforation or having underlying diseases that severely affected the prognosis. We also excluded 28 patients who did not have abdominal CT scans before surgery in our hospital or other necessary clinical data. Finally, this study included a total of 200 patients with upper gastrointestinal perforation, of whom 161 were men. The average age of the patients was 60.2 years. Of these patients, 55 developed septic shock, 50 were transferred to the ICU, and 47 experienced postoperative complications. The study flow diagram is presented in Figure 1.

### 3.2 Risk factors for septic shock

**Figure 2:**
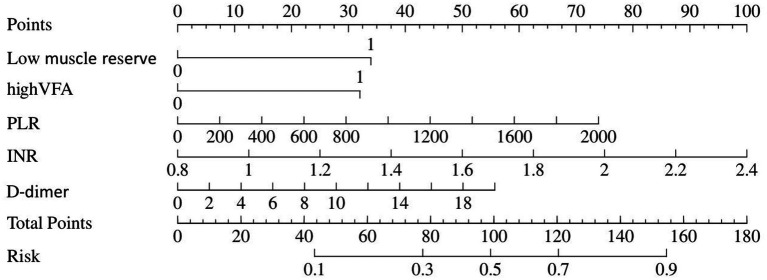
Nomogram represents septic shock.

**Figure 3:**
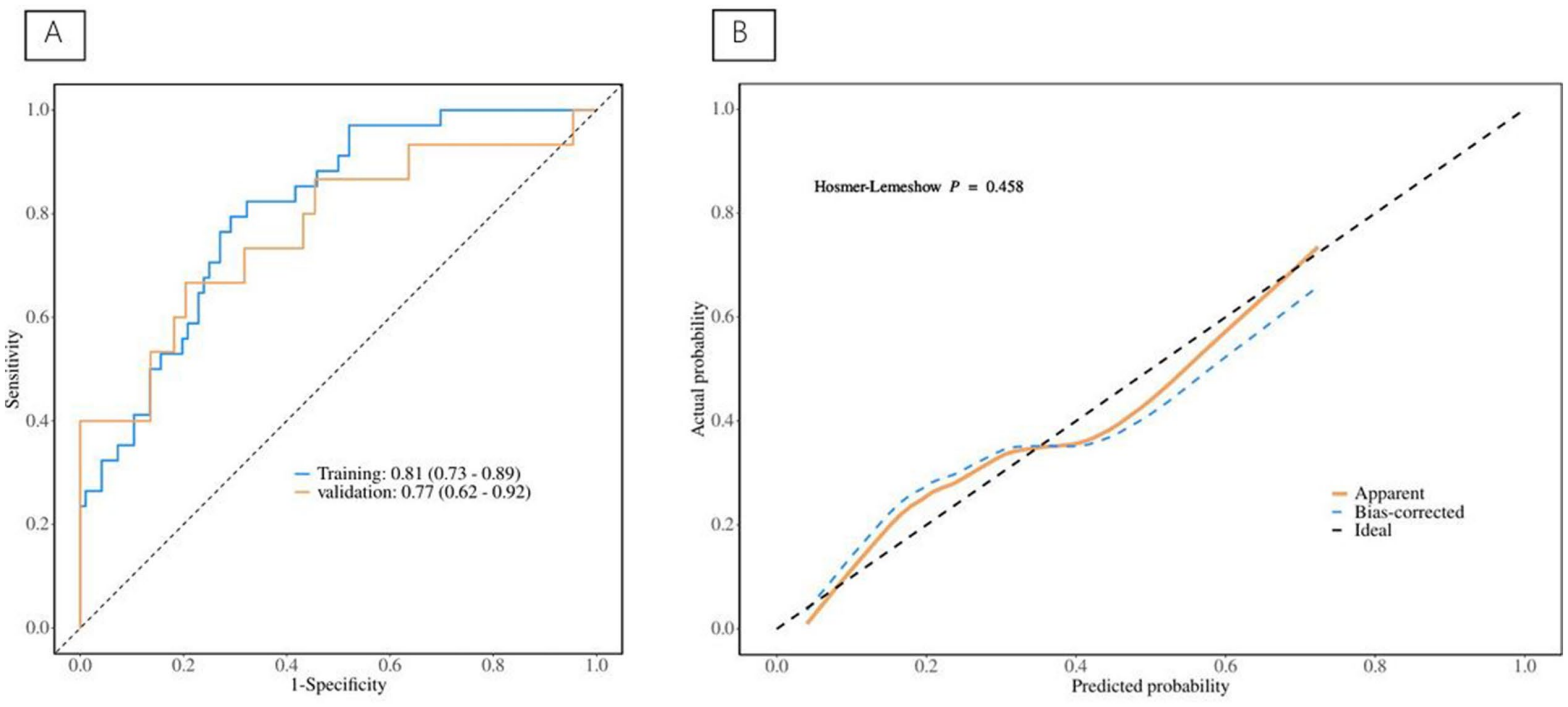
**(A)** The ROC curve for septic shock discrimination. **(B)** The Calibration Curve for septic shock discrimination.

Table 1 depicts information about patients associated with septic shock, and Table 2 shows the predictive model for septic shock. Univariate analyses identified low muscle reserve (*p* < 0.001), high VFA (*p* = 0.036), high VSR (*p* = 0.033), age (*p* = 0.029), PLR (*p* = 0.029), PT (*p* = 0.003), INR (*p* = 0.003), APTT (*p* = 0.039), D-dimer (*p* = 0.012), and CRP (*p* = 0.018) as being potentially independently correlated with septic shock. Multivariate regression analyses were subsequently performed on these factors. Ultimately, the prediction model incorporated low muscle reserve, high VFA, PLR, INR, and D-dimer. Low muscle reserve, high VFA, and PLR were identified as risk factors for septic shock with OR values of 3.82 (95% CI 1.36–10.71, *p* = 0.011), 3.54 (95% CI 1.16–10.80, *p* = 0.026), and 1.01 (95% CI 1.01–1.01, *p* = 0.048), respectively. Figure 2 shows the risk factor nomogram of septic shock for patients with upper gastrointestinal perforation. Figure 3 depicts the ROC curve and the calibration curve for discriminating septic shock, with a C-index of 0.81. This model has a sensitivity of 0.71 (0.62–0.80) and a specificity of 0.79 (0.66–0.93).

**Table 1:** Included patients’ information classified by septic shock.

Variables	No septic shock (*N* = 145)	Septic shock (*N* = 55)	*p*-value
Age (years)	58.4 ± 15.8	65.0 ± 15.4	0.010
Sex			0.005
Female	21 (14.5%)	18 (32.7%)	
Male	124 (85.5%)	37 (67.3%)	
Perforation site			0.716
Stomach	67 (46.2%)	22 (40.0%)	
Duodenum	75 (51.7%)	32 (58.2%)	
Other	3 (2.1%)	1 (1.8%)	
Maximum perforation diameter (mm)	5 (4, 10)	8 (5, 15)	0.038
BMI (kg/m^2^)	20.8 ± 3.0	21.2 ± 4.1	0.381
Hypertension			0.228
Yes	40 (27.6%)	20 (36.4%)	
No	105 (72.4%)	35 (63.6%)	
Diabetes			0.255
Yes	13 (9.0%)	8 (14.5%)	
No	132 (91.0%)	47 (85.5%)	
Heart disease			0.476
Yes	9 (6.2%)	2 (3.6%)	
No	136 (93.8%)	53 (96.4%)	
Low muscle reserve			<0.001
Yes	54 (37.2%)	38 (69.1%)	
No	91 (62.8%)	17 (30.9%)	
Low SFA			0.557
Yes	43 (29.7%)	14 (25.5%)	
No	102 (70.3%)	41 (74.5%)	
High VFA			0.007
Yes	24 (16.6%)	19 (34.5%)	
No	121 (83.4)	36 (65.5%)	
High VSR			0.099
Yes	38 (26.2%)	21 (38.2%)	
No	107 (73.8%)	34 (61.8%)	
WBC (*10^9^/L)	12.1 (9.4, 15.5)	9.9 (5.1, 14.1)	0.064
RBC (*10^12^/L)	4.5 ± 0.7	4.4 ± 1.2	0.560
PLT (*10^9^/L)	234 (196, 286)	277 (169, 354)	0.061
PLR	263.8 (188.4, 437.6)	395.0 (252.6, 647.4)	0.015
NLR	11.6 (7.8, 17.9)	10.8 (4.8, 10.0)	0.753
SII	2722.5 (1755.2, 4742.1)	2565.4 (1211.4, 6223.5)	0.497
CRP (mg/L)	7.1 (1.9, 59.8)	84.0 (26.3, 172.6)	0.002
PT(s)	13.4 (12.9, 14.4)	14.6 (13.7, 15.7)	<0.001
INR	1.02 (0.98–1.12)	1.13 (1.07, 1.25)	<0.001
FIB (g/L)	3.6 (2.9, 4.6)	4.0 (3.2, 5.6)	0.181
APTT(s)	33.3 (30.5, 37.6)	36.3 (32.2, 42.4)	0.011
D-dimer (mg/L)	0.96 (0.51, 2.29)	2.91 (1.74, 4.97)	<0.001
Anti-infection			0.500
Yes	123 (84.8%)	49 (89.1%)	
No	21 (14.5%)	6 (10.9%)	
Time from onset to anti-infection (h)	6.0 (4.0, 11.0)	8.0 (5.0, 17.0)	0.059
Time from onset to surgery (h)	15.0 (10.0, 30.0)	18.8 (13.1, 24.5)	0.630

**Table 2:** Clinical prediction model for septic shock using backward stepwise logistic regression.

Variables	Univariate analysis					Multivariate analysis				
	*β*	S.E.	*Z*	*p*	OR (95% CI)	*β*	S.E.	*Z*	*p*	OR (95% CI)
Sex										
Female					1.00 (Reference)					
Male	−0.81	0.45	−1.79	0.073	0.44 (0.18–1.08)					
Perforation site										
Stomach					1.00 (Reference)					
Duodenum	0.03	0.39	0.08	0.937	1.03 (0.48–2.20)					
Other	−0.13	1.19	−0.11	0.911	0.88 (0.08–9.04)					
Hypertension										
No					1.00 (Reference)					
Yes	0.43	0.40	1.06	0.287	1.53 (0.70–3.38)					
Diabetes										
No					1.00 (Reference)					
Yes	0.29	0.58	0.50	0.618	1.34 (0.43–4.20)					
Heart disease										
No					1.00 (Reference)					
Yes	−0.16	0.84	−0.19	0.853	0.86 (0.17–4.43)					
Low muscle reserve										
No					1.00 (Reference)					1.00 (Reference)
Yes	1.57	0.42	3.74	**<0.001**	4.81 (2.11–10.96)	1.34	0.53	2.55	**0.011**	3.82 (1.36–10.71)
Low SFA										
No					1.00 (Reference)					
Yes	−0.63	0.45	−1.40	0.160	0.53 (0.22–1.28)					
High VFA										
No					1.00 (Reference)					1.00 (Reference)
Yes	0.90	0.43	2.10	**0.036**	2.47 (1.06–5.75)	1.26	0.57	2.22	**0.026**	3.54 (1.16–10.80)
High VSR										
No					1.00 (Reference)					
Yes	0.86	0.40	2.13	**0.033**	2.36 (1.07–5.20)					
Anti-infection										
No					1.00 (Reference)					
Yes	0.18	0.51	0.34	0.732	1.19 (0.44–3.27)					
Time from onset to surgery										
<24 h					1.00 (Reference)					
≥24 h	−0.09	0.39	−0.23	0.817	0.91 (0.43–1.95)					
Age (years)	0.03	0.01	2.19	**0.029**	1.03 (1.01–1.06)					
Maximum perforation diameter (mm)	0.04	0.02	1.80	0.072	1.04 (1.00–1.09)					
BMI (kg/m^2^)	0.07	0.06	1.21	0.226	1.07 (0.96–1.20)					
WBC (*10^9^/L)	−0.02	0.03	−0.72	0.474	0.98 (0.91–1.04)					
RBC (*10^12^/L)	−0.01	0.22	−0.03	0.976	0.99 (0.64–1.54)					
PLT (*10^9^/L)	0.00	0.00	1.43	0.154	1.00 (1.00–1.01)					
PLR	0.01	0.00	2.18	**0.029**	1.01 (1.01–1.01)	0.01	0.00	1.97	**0.048**	1.01 (1.01–1.01)
NLR	0.01	0.01	0.76	0.449	1.01 (0.98–1.04)					
PT(s)	0.37	0.13	2.98	**0.003**	1.45 (1.14–1.86)					
INR	3.72	1.24	2.99	**0.003**	41.44 (3.62–474.44)	2.47	1.37	1.80	0.071	11.79 (0.81–172.08)
FIB (g/L)	0.09	0.11	0.76	0.450	1.09 (0.87–1.36)					
APTT(s)	0.06	0.03	2.06	**0.039**	1.06 (1.01–1.12)					
D-dimer (mg/L)	0.15	0.06	2.52	**0.012**	1.16 (1.03–1.30)	0.11	0.06	1.74	0.081	1.12 (0.99–1.26)
CRP (mg/L)	0.01	0.00	2.37	**0.018**	1.01 (1.01–1.01)					
SII	0.00	0.00	1.38	0.168	1.00 (1.00–1.00)					
Time from onset to anti-infection (h)	0.01	0.01	1.02	0.308	1.01 (0.99–1.02)					
Time from onset to surgery (h)	−0.00	0.00	−0.53	0.594	1.00 (0.99–1.01)					

OR, odds ratio; CI, confidence interval. The bold values represent *p* ≤ 0.05.

### 3.3 Risk factors for ICU admission

**Figure 4:**
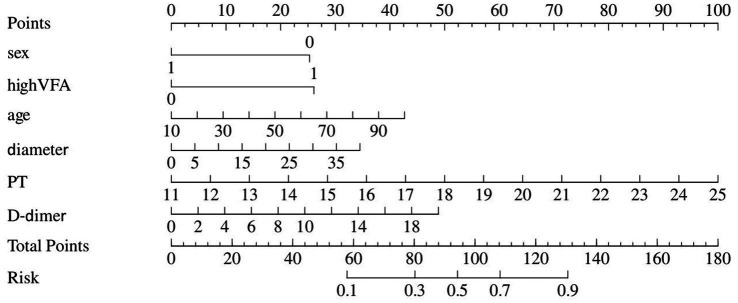
Nomogram represents ICU admission.

**Figure 5:**
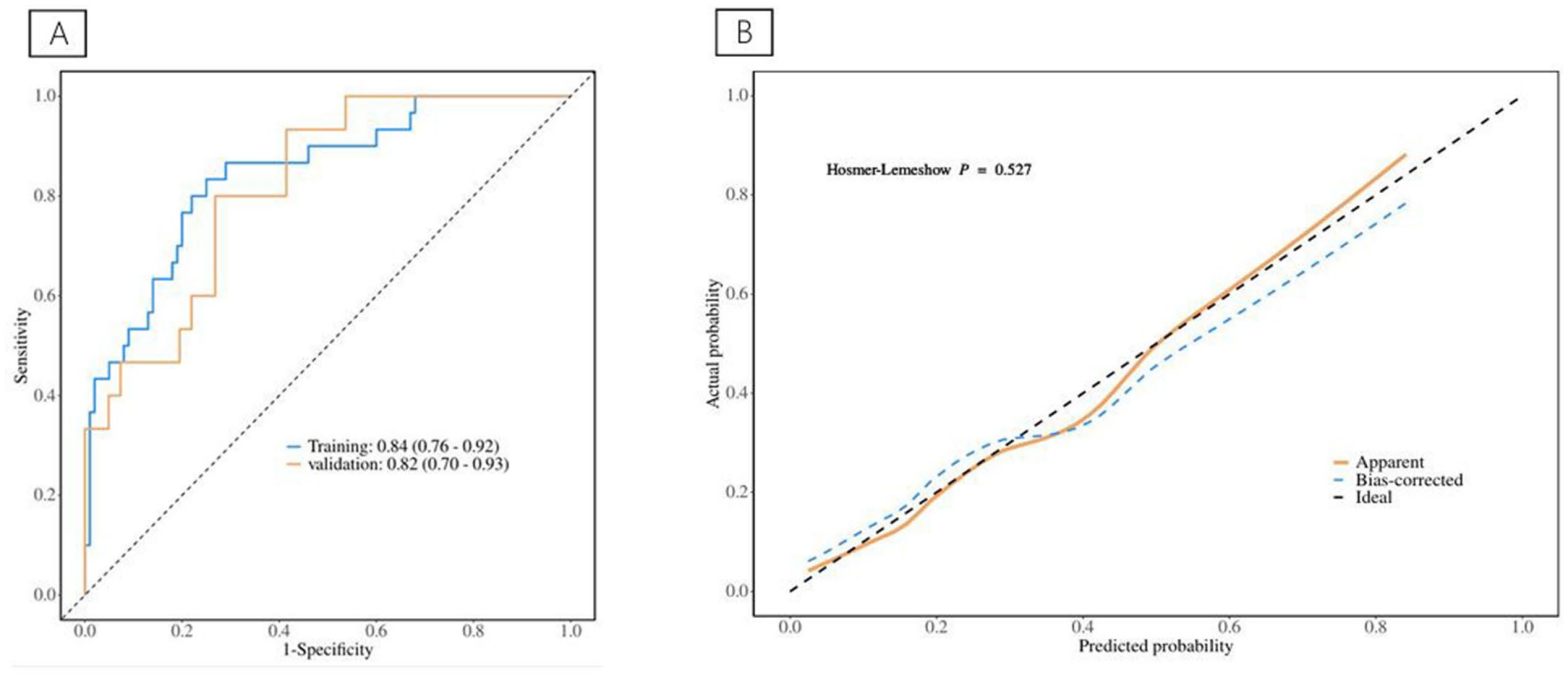
**(A)** The ROC curve for ICU admission discrimination. **(B)** The Calibration Curve for ICU admission discrimination.

Information on patients associated with ICU admission is depicted in Table 3, and the predictive model for ICU admission is depicted in Table 4. Univariate analyses identified variables with a potential independent correlation with ICU admission, revealing that the following were associated with ICU admission: sex (*p* = 0.010), surgical approach (*p* = 0.002), high VFA (*p* = 0.008), age (*p* < 0.001), maximum perforation diameter (*p* = 0.010), PT (*p* < 0.001), INR (*p* < 0.001), D-dimer (*p* = 0.007), and CRP (*p* = 0.047). Multivariate regression analyses were subsequently performed on the aforementioned factors. Ultimately, sex, high VFA, age, maximum perforation diameter, PT, and D-dimer were incorporated into the prediction model, among which sex, high VFA, PT, and D-dimer were identified as risk factors for ICU admission, with OR values of 0.22 (95% CI 0.06–0.78, *p* = 0.020), 4.84 (95% CI 1.38–17.02, *p* = 0.014), 1.54 (95% CI 1.09–2.18, *p* = 0.014), and 1.16 (95% CI 1.01–1.34, *p* = 0.048), respectively. The ICU admission nomogram for patients with upper gastrointestinal perforation is presented in Figure 4. The ROC curve and the calibration curve for distinguishing ICU admission are plotted in Figure 5, and the C-index is 0.84. The sensitivity of this model is 0.75 (0.67–0.83), and the specificity is 0.83 (0.70–0.97).

**Table 3:** Included patients’ information classified by ICU admission.

Variables	No ICU (*N* = 150)	ICU (*N* = 50)	*p*-value
Age (years)	57.5 ± 15.6	68.3 ± 14.5	<0.001
Sex			<0.001
Female	19 (12.7%)	20 (40.0%)	
Male	131 (87.3%)	30 (60.0%)	
Perforation site			0.562
Stomach	70 (46.7%)	19 (38.0%)	
Duodenum	77 (51.3%)	30 (60.0%)	
Other	3 (2.0%)	1 (2.0%)	
Maximum perforation diameter (mm)	5.0 (4.0, 10.0)	10.0 (5.0, 15.0)	0.010
BMI (kg/m^2^)	20.7 ± 3.2	21.4 ± 3.6	0.181
Hypertension			0.077
Yes	40 (26.7%)	20 (40.0%)	
No	110 (73.3%)	30 (60.0%)	
Diabetes			0.894
Yes	16 (10.7%)	5 (10.0%)	
No	134 (89.3%)	45 (90.0%)	
Heart disease			0.612
Yes	9 (6.0%)	2 (4.0%)	
No	141 (94.0%)	48 (96.0%)	
Surgical approach			0.001
Laparoscopic	86 (57.3%)	15 (30.0%)	
Open	64 (42.7%)	35 (70.0%)	
Low muscle reserve			0.010
Yes	61 (40.7%)	31 (62.0%)	
No	89 (59.3%)	19 (38.0%)	
Low SFA			0.242
Yes	46 (30.7%)	11 (22.0%)	
No	104 (69.3%)	39 (78.0%)	
High VFA			0.005
Yes	25 (16.7%)	18 (36.0%)	
No	125 (83.3%)	32 (64.0%)	
High VSR			0.130
Yes	40 (26.7%)	19 (38.0%)	
No	110 (73.3%)	31 (62.0%)	
WBC (*10^9^/L)	12.0 (8.9, 15.5)	10.3 (5.1, 15.6)	0.482
RBC (*10^12^/L)	4.5 ± 0.7	4.2 ± 1.2	0.040
PLT (*10^9^/L)	241.0 (197.5, 282.3)	265.5 (166.5, 359.0)	0.293
PLR	301.6 (190.0, 455.4)	290.0 (202.0, 615.4)	0.146
NLR	11.3 (7.4, 18.3)	11.6 (7.3, 17.6)	0.665
SII	2677.0 (1789.2, 4882.9)	3811.9 (1209.9, 6247.7)	0.743
CRP (mg/L)	7.4 (1.9, 59.8)	79.8 (16.1, 172.6)	0.002
PT(s)	13.4 (12.9, 14.4)	14.6 (13.7, 16.3)	<0.001
INR	1.02 (0.98, 1.12)	1.14 (1.07, 1.30)	<0.001
FIB (g/L)	3.6 (2.9, 4.7)	3.9 (3.1, 5.3)	0.788
APTT(s)	33.3 (30.5, 37.6)	36.6 (32.7, 43.4)	0.003
D-dimer (mg/L)	1.1 (0.5, 2.4)	3.0 (1.8, 4.8)	0.001
Anti-infection			0.709
Yes	128 (85.3%)	44 (88.0%)	
No	21 (14.0%)	6 (12.0%)	
Time from onset to anti-infection (h)	6.0 (4.0, 11.0)	8.0 (5.0, 14.9)	0.274
Time from onset to surgery (h)	15.5 (10.0, 29.0)	18.8 (12.6, 27.8)	0.685

The bold values represent *p* ≤ 0.05.

**Table 4:** Clinical prediction model for ICU admission using backward stepwise logistic regression.

Variables	Univariate analysis					Multivariate analysis				
	*β*	S.E.	*Z*	*p*	OR (95% CI)	*β*	S.E.	*Z*	*p*	OR (95% CI)
Sex										
Female					1.00 (Reference)					1.00 (Reference)
Male	−1.20	0.47	−2.57	**0.010**	0.30 (0.12–0.75)	−1.53	0.66	−2.33	**0.020**	0.22 (0.06–0.78)
Perforation site										
Stomach					1.00 (Reference)					
Duodenum	0.68	0.42	1.63	0.103	1.98 (0.87–4.51)					
Other	1.59	1.45	1.10	0.273	4.91 (0.28–84.58)					
Hypertension										
No					1.00 (Reference)					
Yes	0.76	0.42	1.79	0.074	2.13 (0.93–4.89)					
Diabetes										
No					1.00 (Reference)					
Yes	−1.08	1.07	−1.00	0.315	0.34 (0.04–2.79)					
Heart disease										
No					1.00 (Reference)					
Yes	0.27	0.86	0.32	0.750	1.32 (0.24–7.12)					
Approach										
Laparoscopic					1.00 (Reference)					
Open	1.42	0.45	3.15	**0.002**	4.14 (1.71–10.02)					
Low muscle reserve										
No					1.00 (Reference)					
Yes	0.74	0.40	1.84	0.066	2.10 (0.95–4.64)					
Low SFA										
No					1.00 (Reference)					
Yes	−0.74	0.50	−1.49	0.136	0.48 (0.18–1.26)					
High VFA										
No					1.00 (Reference)					1.00 (Reference)
Yes	1.17	0.44	2.65	**0.008**	3.21 (1.36–7.62)	1.58	0.64	2.46	**0.014**	4.84 (1.38–17.02)
High VSR										
No					1.00 (Reference)					
Yes	0.76	0.41	1.85	0.064	2.14 (0.96–4.78)					
Anti-infection										
No					1.00 (Reference)					
Yes	−0.16	0.56	−0.28	0.777	0.85 (0.28–2.57)					
Time from onset to surgery										
<24 h					1.00 (Reference)					
≥24 h	0.54	0.40	1.33	0.183	1.71 (0.78–3.76)					
Age (years)	0.05	0.01	3.30	**<0.001**	1.05 (1.02–1.08)	0.03	0.02	1.66	0.098	1.03 (0.99–1.06)
Maximum perforation diameter (mm)	0.08	0.03	2.59	**0.010**	1.08 (1.02–1.14)	0.05	0.03	1.54	0.124	1.05 (0.99–1.13)
BMI (kg/m^2^)	0.05	0.06	0.81	0.420	1.05 (0.94–1.17)					
WBC (*10^9^/L)	0.05	0.03	1.35	0.177	1.05 (0.98–1.12)					
RBC (*10^12^/L)	−0.42	0.24	−1.77	0.077	0.66 (0.41–1.05)					
PLT (*10^9^/L)	0.00	0.00	1.95	0.052	1.00 (1.00–1.01)					
PLR	0.00	0.00	0.81	0.417	1.00 (1.00–1.00)					
NLR	0.00	0.02	0.11	0.915	1.00 (0.97–1.03)					
PT(s)	0.53	0.15	3.46	**<0.001**	1.70 (1.26–2.29)	0.43	0.18	2.44	**0.014**	1.54 (1.09–2.18)
INR	5.04	1.49	3.37	**<0.001**	154.28 (8.24–2888.72)					
FIB (g/L)	0.00	0.13	0.01	0.992	1.00 (0.77–1.29)					
APTT(s)	0.05	0.03	1.67	0.095	1.05 (0.99–1.11)					
D-dimer (mg/L)	0.17	0.06	2.69	**0.007**	1.19 (1.05–1.34)	0.15	0.07	1.98	**0.048**	1.16 (1.01–1.34)
CRP (mg/L)	0.01	0.00	1.99	**0.047**	1.01 (1.01–1.01)					
SII	0.00	0.00	0.84	0.402	1.00 (1.00–1.00)					
Time from onset to anti-infection (h)	0.01	0.01	0.97	0.330	1.01 (0.99–1.02)					
Time from onset to surgery (h)	−0.00	0.00	−0.20	0.839	1.00 (0.99–1.01)					

OR, odds ratio; CI, confidence interval. The bold values represent *p* ≤ 0.05.

### 3.4 Risk factors for postoperative complications

**Figure 6:**
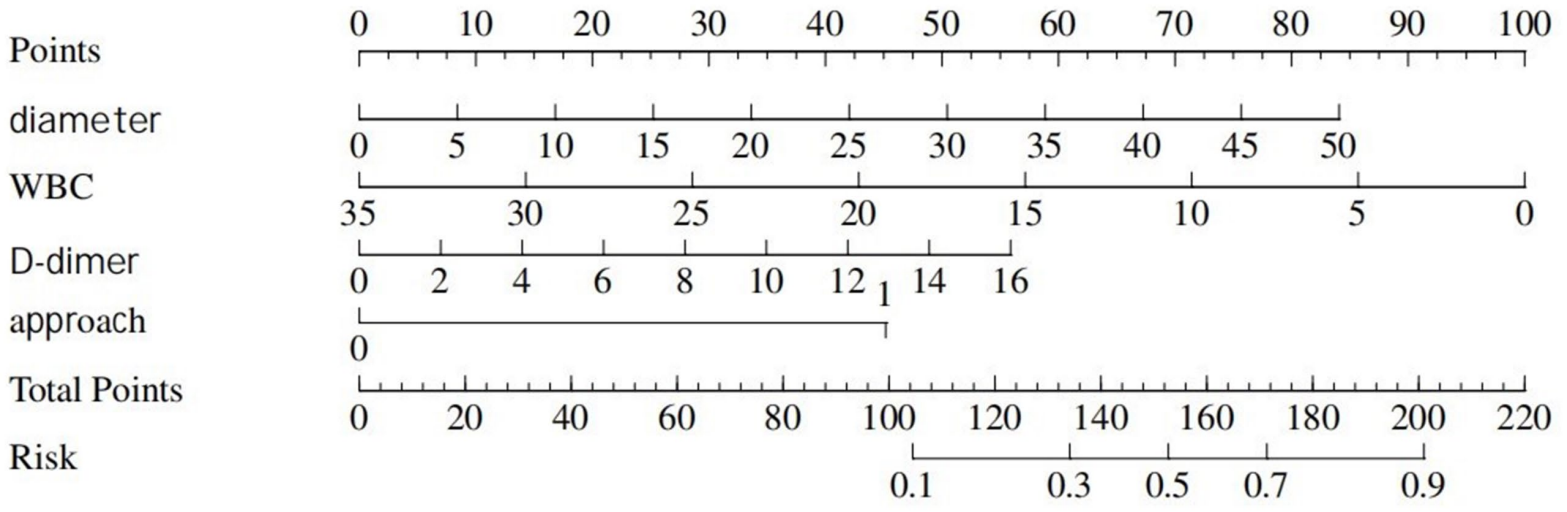
Nomogram represents postoperative complications.

**Figure 7:**
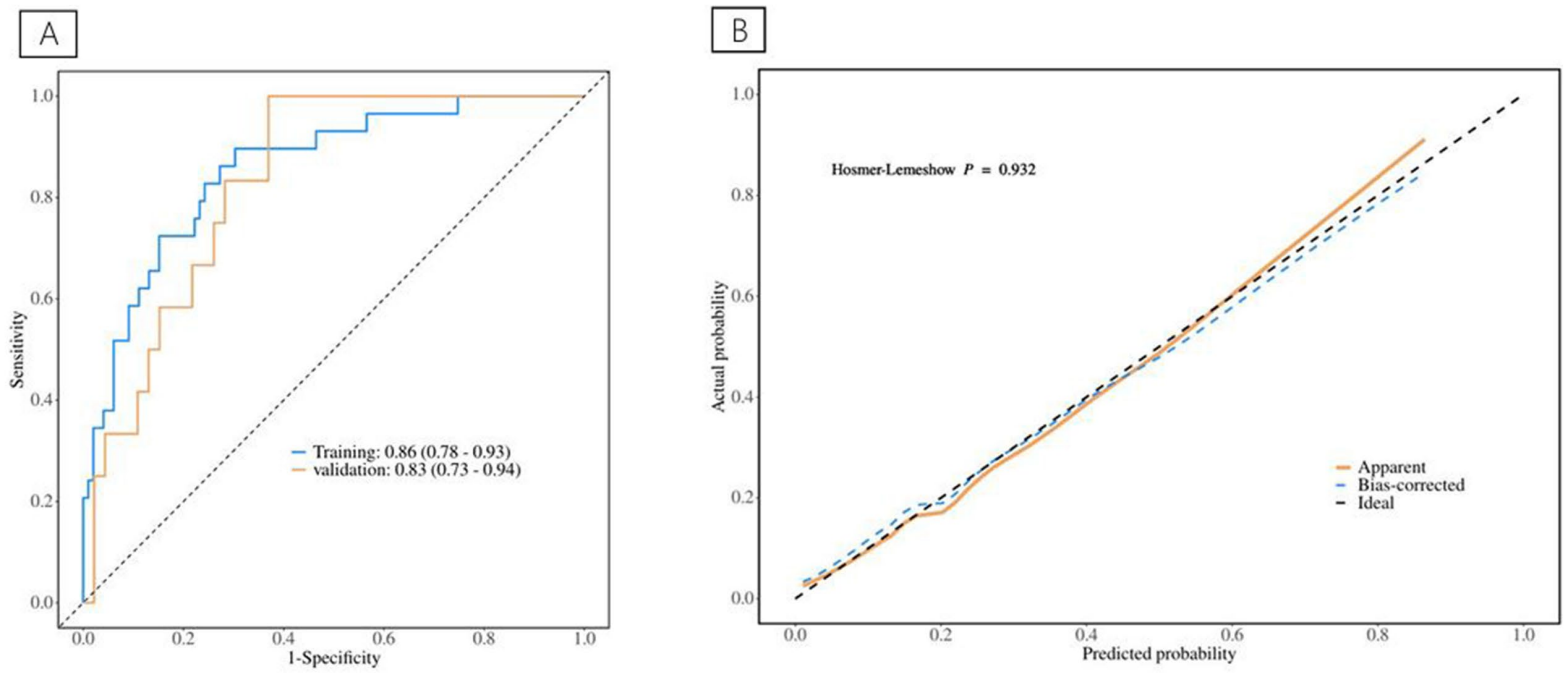
**(A)** The ROC curve for postoperative complications discrimination. **(B)** The Calibration Curve for postoperative complications discrimination.

Table 5 displays information about patients who have experienced postoperative complications, and Table 6 displays the predictive model for postoperative complications. Univariate analyses identified variables with a potential independent correlation with postoperative complications, indicating the following: sex (*p* = 0.027), hypertension (*p* = 0.013), surgical approach (*p* < 0.001), low muscle reserve (*p* = 0.007), age (*p* = 0.018), maximum perforation diameter (*p* < 0.001), WBC (*p* = 0.014), PLT (*p* = 0.038), FIB (*p* = 0.009), D-dimer (*p* < 0.001), CRP (*p* = 0.006), and time from onset to anti-infection (*p* = 0.046). Multivariate regression analyses were subsequently performed on the aforementioned factors. Ultimately, surgical approach, maximum perforation diameter, WBC, and D-dimer were incorporated into the prediction model, among which the surgical approach, the maximum perforation diameter and the WBC count were identified as risk factors for postoperative complications, with OR values of 7.82 (95% CI 1.94–31.57, *p* = 0.004), 1.08 (95% CI 1.02–1.15, *p* = 0.013), and 0.88 (95% CI 0.78–0.99, *p* = 0.039), respectively. Figure 6 depicts the risk factors nomogram of postoperative complications. Figure 7 depicts the ROC curve and the calibration curve for discriminating postoperative complications, with a C-index of 0.86. This model has a sensitivity of 0.70 (0.61–0.79) and a specificity of 0.90 (0.79–1.00).

**Table 5:** Included patients’ information classified by postoperative complications.

Variables	No postoperative complications (*N* = 153)	Postoperative complications (*N* = 47)	*p*-value
Age (years)	58.5 ± 15.6	65.9 ± 16.1	0.006
Sex			0.016
Female	24 (15.7%)	15 (31.9%)	
Male	129 (84.3%)	32 (68.1%)	
Perforation site			0.207
Stomach	70 (45.8%)	19 (40.4%)	
Duodenum	81 (52.9%)	26 (55.3%)	
Other	2 (1.3%)	2 (4.3%)	
Maximum perforation diameter (mm)	5.0 (4.0, 8.0)	10.0 (5.0, 15.0)	<0.001
BMI (kg/m^2^)	20.9 ± 3.2	20.7 ± 3.6	0.659
Hypertension			0.005
Yes	38 (24.8%)	22 (46.8%)	
No	115 (75.2%)	25 (53.2%)	
Diabetes			0.563
Yes	15 (9.8%)	6 (12.8%)	
No	138 (90.2%)	41 (87.2%)	
Heart disease			0.691
Yes	9 (5.9%)	2 (4.3%)	
No	144 (94.1%)	45 (95.7%)	
Surgical approach			<0.001
Laparoscopic	96 (62.7%)	5 (10.6%)	
Open	57 (37.3%)	42 (89.4%)	
Low muscle reserve			0.006
Yes	62 (40.5%)	30 (63.8%)	
No	91 (59.5%)	17 (36.2%)	
Low SFA			0.607
Yes	45 (29.4%)	12 (25.5%)	
No	108 (70.6%)	35 (74.5%)	
High VFA			0.117
Yes	29 (19.0%)	14 (29.8%)	
No	124 (81.0%)	33 (70.2%)	
High VSR			0.436
Yes	43 (28.1%)	16 (34.0%)	
No	110 (71.9%)	31 (66.0%)	
WBC (*10^9^/L)	12.2 (9.0, 15.8)	10.1 (5.1, 12.5)	0.011
RBC (*10^12^/L)	4.5 ± 0.7	4.3 ± 1.2	0.079
PLT (*10^9^/L)	232.0 (192.5, 280.0)	281.0 (227.0, 345.0)	0.032
PLR	272.8 (188.4, 449.5)	358.4 (249.1, 632.2)	0.069
NLR	11.7 (7.9, 18.7)	8.5 (4.5, 16.5)	0.180
SII	2722.5 (1740.4, 5018.2)	2542.4 (1127.5, 4808.9)	0.888
CRP (mg/L)	7.6 (2.3, 74.3)	67.3 (16.4, 160.7)	0.007
PT(s)	13.4 (12.9, 14.5)	14.4 (13.5, 15.7)	0.005
INR	1.03 (0.98, 1.14)	1.11 (1.04, 1.24)	0.006
FIB (g/L)	3.6 (2.9, 4.6)	4.2 (3.1, 5.9)	0.025
APTT(S)	33.3 (30.0, 38.2)	36.1 (33.0, 38.4)	0.088
D-dimer (mg/L)	1.1 (0.5, 2.5)	2.7 (1.5, 5.0)	0.001
Anti-infection			0.762
Yes	132 (86.3%)	40 (85.1%)	
No	20 (13.1%)	7 (14.9%)	
Time from onset to anti-infection (h)	6.0 (4.0, 9.4)	11.5 (5.0, 24.0)	0.034
Time from onset to surgery (h)	11.5 (10.0, 29.3)	18.3 (12.8, 27.3)	0.864

**Table 6:** Clinical prediction model for postoperative complications using backward stepwise logistic regression.

Variables	Univariate analysis					Multivariate analysis				
	*β*	S.E.	*Z*	*p*	OR (95% CI)	*β*	S.E.	*Z*	*p*	OR (95% CI)
Sex										
Female					1.00 (Reference)					
Male	−0.98	0.44	−2.22	**0.027**	0.38 (0.16–0.89)					
Perforation site										
Stomach					1.00 (Reference)					
Duodenum	−0.01	0.40	−0.02	0.984	0.99 (0.45–2.19)					
Other	1.88	1.26	1.49	0.136	6.53 (0.55–77.17)					
Hypertension										
No					1.00 (Reference)					
Yes	1.00	0.40	2.48	**0.013**	2.72 (1.23–6.01)					
Diabetes										
No					1.00 (Reference)					
Yes	0.40	0.58	0.69	0.492	1.49 (0.48–4.64)					
Heart disease										
No					1.00 (Reference)					
Yes	−0.96	1.08	−0.89	0.374	0.38 (0.05–3.18)					
Approach										
Laparoscopic					1.00 (Reference)					1.00 (Reference)
Open	2.72	0.57	4.77	**<0.001**	15.21 (4.97–46.58)	2.06	0.71	2.89	**0.004**	7.82 (1.94–31.57)
Low muscle reserve										
No					1.00 (Reference)					
Yes	1.12	0.42	2.69	**0.007**	3.06 (1.35–6.93)					
Low SFA										
No					1.00 (Reference)					
Yes	−0.00	0.46	−0.01	0.995	1.00 (0.40–2.48)					
High VFA										
No					1.00 (Reference)					
Yes	0.66	0.44	1.50	0.133	1.94 (0.82–4.59)					
High VSR										
No					1.00 (Reference)					
Yes	0.39	0.43	0.90	0.370	1.47 (0.63–3.42)					
Anti-infection										
No					1.00 (Reference)					
Yes	−0.30	0.57	−0.52	0.600	0.74 (0.24–2.28)					
Time from onset to surgery										
<24 h					1.00 (Reference)					
≥24 h	0.58	0.40	1.46	0.144	1.79 (0.82–3.91)					
Age (years)	0.03	0.01	2.37	**0.018**	1.03 (1.01–1.06)					
Maximum perforation diameter (mm)	0.11	0.03	3.58	**<0.001**	1.11 (1.05–1.18)	0.08	0.03	2.48	**0.013**	1.08 (1.02–1.15)
BMI (kg/m^2^)	−0.02	0.06	−0.32	0.745	0.98 (0.87–1.11)					
WBC (*10^9^/L)	−0.10	0.04	−2.45	**0.014**	0.91 (0.84–0.98)	−0.13	0.06	−2.06	**0.039**	0.88 (0.78–0.99)
RBC (*10^12^/L)	−0.36	0.25	−1.48	0.138	0.69 (0.43–1.12)					
PLT (*10^9^/L)	0.01	0.00	2.07	**0.038**	1.01 (1.01–1.01)					
PLR	0.00	0.00	1.18	0.237	1.00 (1.00–1.00)					
NLR	−0.04	0.02	−1.70	0.088	0.96 (0.92–1.01)					
PT(s)	0.15	0.10	1.49	0.136	1.17 (0.95–1.42)					
INR	1.42	1.01	1.40	0.162	4.12 (0.57–29.95)					
FIB (g/L)	0.30	0.12	2.62	**0.009**	1.36 (1.08–1.70)					
APTT(s)	0.04	0.03	1.29	0.196	1.04 (0.98–1.11)					
D-dimer (mg/L)	0.27	0.07	3.62	**<0.001**	1.31 (1.13–1.51)	0.16	0.10	1.61	0.107	1.17 (0.97–1.42)
CRP (mg/L)	0.01	0.00	2.74	**0.006**	1.01 (1.01–1.01)					
SII	−0.00	0.00	−0.76	0.449	1.00 (1.00–1.00)					
Time from onset to anti-infection (h)	0.01	0.01	2.00	**0.046**	1.01 (1.01–1.03)					
Time from onset to surgery (h)	0.00	0.00	1.23	0.219	1.00 (1.00–1.01)					

OR, odds ratio; CI, confidence interval.

## 4 Discussion

Despite improvements in antibiotic utilization and medical standards, the mortality rate for patients with gastrointestinal perforation remained between 10 and 50% (1–3). This was largely attributed to the development of chemical or bacterial peritonitis induced by perforation. Severe cases might progress to suppurative peritonitis, which could subsequently lead to sepsis or a severe systemic infection, ultimately culminating in shock. This pathological cascade could further induce multiple organ dysfunction syndrome (MODS), endangering patient survival. Consequently, septic shock was a critical factor influencing the prognosis of patients with gastrointestinal perforation, and identifying its contributing factors remained a key area of research.

Poor nutritional status was a risk factor for an adverse prognosis in cases of gastrointestinal perforation (4, 5, 27). Nutritional reserves were essential for defending against bacterial invasion, and patients with low PNI and total protein levels were susceptible to septic shock (5). However, due to the emergent nature of perforation, it was difficult to obtain these nutritional indicators preoperatively. Therefore, body composition parameters were used as an indicator of nutritional status. Our study demonstrated that low muscle reserve and high VFA were risk factors for septic shock. The skeletal muscle system constitutes approximately 40% of the adult body volume, approximately 88% of which is protein, which represents 50% of the body’s total protein content. Muscles act as a nutrient storage system, distributing amino acids to various organs in a biological defense response to invasion. Therefore, the preoperative skeletal muscle mass could serve as a prognostic indicator in cases of upper gastrointestinal perforation. During emergency surgery in the presence of severe infection, a greater baseline muscle mass was more beneficial for tissue repair during the defense response, helping to prevent organ failure. However, even in septic patients with increased body fat, aggressive nutritional support could not prevent the loss of the body’s protein reserves (28). Consequently, preoperative medical interventions were unlikely to ameliorate skeletal muscle loss in patients with upper gastrointestinal perforation. To improve survival rates, early surgical intervention and medication might be necessary in the muscle loss group.

VFA was also a significant indicator of nutritional response. Although numerous studies have examined the influence of visceral fat on survival in patients with malignancies, research on its relationship to upper gastrointestinal perforation is scarce. In our study, high VFA was a risk factor for septic shock. Visceral adipose tissue secreted inflammatory cytokines, such as monocyte chemoattractant protein-1 (MCP-1), interleukin-6 (IL-6), and tumor necrosis factor-alpha (TNF-α), participating in systemic inflammatory responses (29). It could also promote insulin resistance, weakening the body’s anti-infective defenses and inducing mitochondrial dysfunction in sepsis (30). Therefore, excessive visceral fat triggered more intense inflammatory reactions and impaired antimicrobial defense mechanisms, ultimately leading to septic shock. This condition was significantly associated with a poor prognosis.

Numerous studies have linked inflammatory responses to the prognosis of gastrointestinal perforation, indicating that heightened inflammation was closely associated with dysregulated immune responses that could potentially trigger septic shock and adverse outcomes. PLR and NLR were common inflammatory indices. Aydin and Pehlivanlı’s (31) study demonstrated a positive correlation between PLR, NLR, and mortality in peptic ulcer perforation, while a negative correlation was observed with lymphocyte count. Similarly, Shimoyama’s et al. (32) study found that NLR and PLR were independently associated with mortality in cases of gastrointestinal perforation. Conversely, a recent study by Yuan et al. (5) showed significantly elevated PLR and lymphocyte-to-monocyte ratio (LMR) in cases of upper gastrointestinal perforation. Furthermore, the present study found that severe inflammation was related to septic shock in upper gastrointestinal perforation, which is consistent with previous findings. This study identified PLR as an influencing factor for septic shock, whereas NLR showed no correlation, and the underlying mechanisms warrant further investigation.

Certain critically ill patients require admission to the ICU, leading to increased healthcare costs and prolonged LOS. Multiple large-scale meta-analyses have demonstrated that female patients tended to be more severely ill upon ICU admission (33, 34). Sivaram and Sreekumar’s (35) study further confirmed significantly higher mortality rates among female patients than male patients. Similarly, our study identified female gender as a risk factor for ICU admission. High VFA constituted another risk factor for ICU transfer. As previously established, elevated VFA was associated with excessive inflammatory responses, potentially exacerbating clinical conditions and necessitating ICU admission. D-dimer was identified as a predictive factor for ICU admission in our study. Current research reported D-dimer as a predictor of adverse outcomes in critical illnesses such as septic shock and mortality (36, 37). This association was likely mediated by the interplay between D-dimer and inflammatory/coagulation pathways. Finally, we observed that although low muscle reserve was not an independent risk factor for ICU admission, patients in this group had significantly longer ICU LOS and higher APACHE II scores, which were a direct indicator of critical illness severity.

Postoperative complications constituted a critical component of a patient’s prognosis. Our study demonstrated that the surgical approach was a predictor of these postoperative complications. Based on a systematic review and meta-analysis of 1,177 patients with perforated peptic ulcers, Panin et al. (38) demonstrated the significant advantages of laparoscopic perforation repair. Compared with traditional open surgical repair, laparoscopic surgery resulted in shorter hospital stays, lower overall postoperative complication rates, and reduced mortality. These findings were consistent with the results of a retrospective study involving 250 patients with perforations (39). Potential contributing factors include minimized incision trauma (40), reduced physiological stress (41), precise instrument manipulation (42), and diminished visceral irritation (43).

Concurrently, our study identified leukocyte count as a predictor of postoperative complications. As one of the most critical immune cell types in sepsis (44), leukocytosis often indicates severe infection or poor prognosis. However, it was noted that reduced leukocyte counts were associated with an increased risk of postoperative complications in our cohort, which was inconsistent with previous studies. This discrepancy might be due to suppressed leukocyte responses during severe infections or to the confounding effect of preoperative antibiotic administration on the measurement. The underlying mechanisms warrant further investigation.

Additionally, a significant correlation was found between the perforation diameter and postoperative complications. In a prospective observational study of 101 patients with perforations, Sivaram and Sreekumar (35) identified perforation diameters of >1 cm as a significant prognostic factor, while Taş et al. (45) reported an elevated risk of postoperative complications in patients with perforation diameters of >0.5 cm. Collectively, these findings aligned with our results, indicating that perforation diameter served as a predictor of postoperative complications. This correlation might be attributable to larger perforations inducing more severe infection and subsequent inflammatory responses, thereby increasing susceptibility to postoperative complications.

This study constructed three clinical prediction models related to the prognosis of upper gastrointestinal perforation. Compared with previous research, this study was the first to incorporate body composition parameters into model construction. These parameters were more accessible preoperatively than other nutritional indices. Furthermore, the prediction models developed in this study demonstrated comparable predictive performance to those established in other studies (5).

There are several limitations to this study. First, it was a single-center investigation. Second, the retrospective study design meant that data for certain variables were missing. Third, the mean age of the enrolled patients exceeded 60 years, resulting in limited representativeness of the study population. Despite receiving standardized training, the researchers involved exhibited a degree of subjectivity during the identification and measurement of body composition parameters, which could have introduced measurement bias. Finally, the predictive models in this study only underwent internal validation. In the future, we will conduct a large-scale, multicenter study to validate these conclusions.

In summary, the key factors influencing the prognosis of upper gastrointestinal perforation were low muscle mass, high VFA, D-dimer levels, PT, PLR, sex, surgical approach, WBC count, and perforation size. These parameters warrant particular attention in clinical practice to identify cases at risk of adverse outcomes.

## Data Availability

The raw data supporting the conclusions of this article will be made available by the authors, without undue reservation.
